# The use of antiviral Phthalocyanine mouthwash as a preventive measure against COVID-19

**DOI:** 10.3205/dgkh000395

**Published:** 2021-07-09

**Authors:** Fabiano Vieira Vilhena, Verônica Caroline Brito Reia, Bernardo da Fonseca Orcina, Caíque Andrade Santos, Mariana Zangrando, Rodrigo Cardoso de Oliveira, Paulo Sérgio da Silva Santos

**Affiliations:** 1 TRIALS – Oral Health & Technologies, Bauru, São Paulo, Brazil; 2 Bauru School of Dentistry, University of São Paulo, Bauru, São Paulo, Brazil

## Letter to the editor

Vaccines and other drugs have been developed and delivered to fight SARS-CoV-2. Successful measures to prevent infection with the virus, such as wearing masks, attention to hygiene and social distancing in conjunction with antiviral mouthwashes, have been recommended to the population for daily use during the COVID-19 pandemic [1], [2].

**Figure 1:**
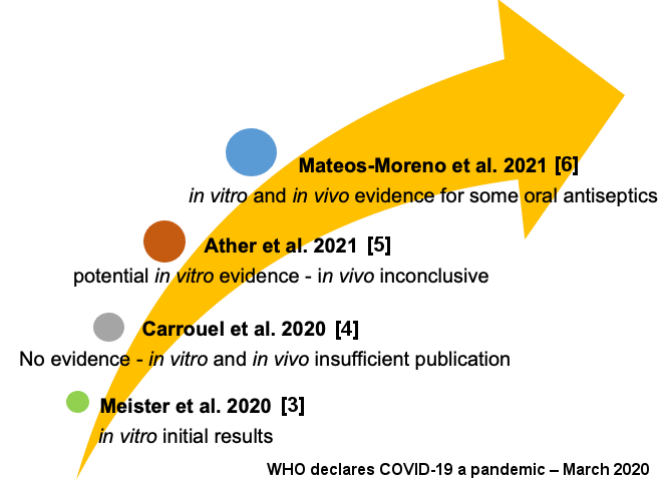
Timeline of scientific evidence for antiviral mouthwash recommendation against SARS-CoV-2

Scientific evidence has shown the presence of viruses in oral structures and indications for antiviral oral antiseptics have been investigated. Thus, *in vitro* studies with mouthwashes against coronavirus published during the pandemic seemed promising [3]. However, until September 2020 there was no clinical or laboratorial evidence for their indication, since only few studies had been published [4]. In March 2021 (Ather et al. 2021 [5]), based on the available evidence. It was demonstrated that the use of antiviral oral antiseptics (mouthrinses) had the potential to reduce the *in vitro* viral load of SARS-CoV-2, but the *in vivo* effectiveness was still inconclusive [5]. Only in April 2021 was it suggested that there was sufficient in vitro and in vivo evidence of the effectiveness of some oral antiseptics in inactivating SARS-CoV-2 and other coronaviruses [6] (Figure 1 (Fig. 1)).

Our research group reinforces the claims for the potential of antiviral mouthwashes to combat COVID-19. Recently, an *in vitro* evaluation of the virucidal activity of a mouthwash containing antiviral phthalocyanine derivative (APD), a substance that can promote reactive oxygen species generation or redox processes [7], was conducted according to the TCID50 methodology (Median Tissue Culture Infectious Dose). The periods of 30 seconds, 1 minute, and 5 minutes were observed, reaching a percentage of viral inactivation above 99.9% of the SARS-CoV-2 viral load, as shown in Table 1 (Tab. 1). This research was approved by Human Research Ethics Committee (CAAE 34070620.6.0000.5417) of the institution that performed the research.

This is another relevant result of the positive action of APD, since a reduction in the viral load of SARS-CoV-2 above 90% has already been demonstrated in previous *in vitro* studies [8], [9], as well as the rapid clinical improvement of COVID-19 symptoms [10]. In a randomized study conducted in a hospital environment, COVID-19 patients with mild and moderate cases underwent an adjuvant APD mouthwash protocol. The APD mouthwash group, with a maximum of 7 days of symptoms associated with conventional treatment for COVID-19, was discharged sooner (4 days median). This group also presented lower disease severity (without intensive care unit [ICU] or deaths) when compared to the non-APD mouthwash group (7 days median, 28.6% ICU, 14.3% mortality rate) [8].

In short, the prospect of a low-cost, hygienic personal-care method, suggestive of good option such as the use of antiviral oral antiseptic, may be an important strategy for reducing the impact of COVID-19.

## Notes

### Competing interests

All authors submitted the ICMJE Form for Disclosure of Potential Conflicts of Interest. Dr. Vilhena reports personal fees from TRIALS Inc while conducting the study. In addition, Dr. Vilhena has a patent pending. Dr. DA Silva Santos reports grants from CNPq process nº. 309525/2018-7. The other authors claim no conflicts of interest

### Funding

This study was financed in part by the Coordenação de Aperfeiçoamento de Pessoal de Nível Superior (CAPES), Brazil (Finance Code 001).

### Acknowledgments

CROP Biotechnology for the experimental design and execution. 

## Figures and Tables

**Table 1:**
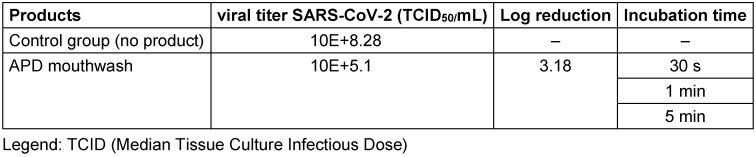
Percentage of viral inactivation of APD Mouthwash
